# Spontaneously occurring cardiovascular lesions in commonly used laboratory animals

**DOI:** 10.1186/s40959-019-0040-y

**Published:** 2019-06-03

**Authors:** Eugene Herman, Sandy Eldridge

**Affiliations:** Division of Cancer Treatment and Diagnosis, National Cancer Institute, Bethesda, Maryland 20892 USA

**Keywords:** Antineoplastic cardiotoxicity, Pathology, Rat, Mouse, Dog, Monkey, Pig

## Abstract

The search for new chemical entities which are clinically effective and do not adversely affect the cardiovascular system is an ongoing objective. In vivo studies designed to detect potential drug-induced cardiovascular toxicity typically utilize both rodent and non-rodent species. An important component of such studies includes the microscopic evaluation of tissues for histopathologic changes. A factor which could potentially complicate this type of evaluation relates to the potential for laboratory animals to develop natural or spontaneous pathological cardiovascular lesions. Some types of these naturally occurring alterations are similar to those induced by chemical compounds and thus could confound accurate interpretation. Accurate morphologic analysis becomes contingent upon the ability to distinguish spontaneous cardiovascular changes from actual drug-induced lesions. A summary of some of the more frequently reported spontaneous cardiovascular alterations in commonly-used laboratory animals is presented below. Special emphasis is given to the spectrum of spontaneous background myocardial pathology that might be encountered during preclinical studies conducted to identify potential cardiotoxic actions of anticancer agents.

## Background

The initial search for new, effective, and safe therapeutic agents involves the evaluation of drug activity in various animal models. Preclinical studies, as part of this process, have played an important role in identifying and elaborating the characteristics of drug-induced toxicity. These studies, undertaken in multiple species, include a portion that is designed to detect an agent’s potential for exerting adverse cardiovascular toxicity. Cardiotoxicity has been identified as a major adverse dose-limiting side effect associated with a number of chemotherapeutic agents. The first reports indicating that cardiotoxicity could pose a serious clinical problem to patients undergoing chemotherapy were reported with the anthracycline antineoplastic agents daunorubicin [1] and doxorubicin [2]. Initially, anthracyclines were responsible for the majority of chemotherapy-induced cardiotoxic reports. However, over the years additional research has led to an increase in the number and types of antineoplastic agents available for use (monoclonal antibodies, immune checkpoint inhibitors, protein kinase inhibitors). Correspondingly, many of these agents have been associated with chemotherapy-related adverse cardiovascular effects [3–6]. A variety of chemotherapeutic compounds have also been found to induce cardiac alterations in preclinical studies (Table 1).

**Table 1:** Cardiotoxic Effects of Selected Chemotherapeutic Agents in Animals

Drug	Class	Types of Alterations	Animal Model	Reference
Lapatinib	Protein kinase inhibitor	Myocardial necrosis	C57BL/6 mouse	[7]
Nilotinib	Tyrosine kinase inhibitor	Increased heart weight	Sprague-Dawley rat	[8]
Imatinib	Tyrosine kinase inhibitor	Cytoplasmic vacuolization, myofibrillar loss, necrosis	Sprague-Dawley rat	[9]
Sunitinib	Tyrosine kinase inhibitor	Capillary proliferation, vacuolization, pericardial inflammation, age-related increased myocyte size	Sprague-Dawley rat	[10]
Sunitinib	Tyrosine kinase inhibitor	Mild vascular congestion	ICR mouse	[11]
Cyclosporine A	Immunosuppressant	Myocardial edema, inflammation, disorganization, necrosis	Sprague-Dawley rat	[12]
Doxorubicin	Anthracycline	Atrial thrombosis, myocyte vacuolization and degeneration; interstitial inflammation	ICR mouse Wistar rat Beagle dog New Zealand rabbit Miniature pig	[13–16]
Sorafenib	Tyrosine kinase inhibitor	Swollen vacuolated myocytes; myofibrillar disorganization	Mouse	[17]
Cisplatin	Inorganic platinum complex	Decreased heart weight, enhanced angiogenesis	C57 mouse	[18]
Cisplatin	Inorganic platinum complex	Myocyte necrosis and cytoplasmic vacuolization; hemorrhage, interstitial edema	Wistar rat	[19, 20]
Cyclophosphamide	Alkylating agent	Interstitial myocardial hemorrhage, multifocal myofiber necrosis, inflammation, vascular endothelial damage, pericarditis, valvulitis	Rat	[21, 22]
Cyclophosphamide	Alkylating agent	Hemorrhagic myocarditis	Rabbit	[23]
5-Fluorouracil	Antimetabolite	Interstitial hemorrhage, multifocal myocyte necrosis, inflammation, small blood vessel inflammation, valvulitis, pericarditis	Rat	[24]
5-Fluorouracil	Antimetabolite	Chronic left ventricular hypertrophy, myocardial necrosis, thickening intra-myocardial arterioles; acute hemorrhagic myocardial infarct, spasms of proximal coronary arteries	Rabbit	[25]
Interleukin-2	T cell growth factor	Endothelial cell hypertrophy and hyperplasia, perivascular inflammation, myocardial necrosis	Wistar rat	[26]
Vincristine	Tubulin binding	Diffuse ventricular myocyte degeneration and necrosis; vacuoles and eosinophilic granules present in degenerating myocytes	Sprague-Dawley rat	[27]
Carfilzomib	Protease inhibitor	Myocardial degeneration, myofibrillar cytoplasmic vacuoles, bands of hypochromatic cells with pyknotic nuclei, inflammation	Rat	[28]

Even though the animals usually selected to be used in preclinical studies are young and healthy, there are reports that indicate some of these normal nontreated animals are prone to develop abnormal cardiovascular alterations. Such spontaneously occurring pathological lesions may confound cardiovascular safety assessments [29, 30]. In such cases, accurate pathologic analysis becomes contingent on the ability to distinguish spontaneous cardiovascular changes from actual drug-induced lesions. Some lesions might mimic those induced by chemical compounds and thus might compromise the interpretation of cardiotoxicity studies, particularly if the lesions are similar to those induced by test agents. An example of a possible spontaneous lesion complication is rodent progressive cardiomyopathy, which occurs spontaneously in young and old animals and that may be exacerbated by drug treatment or even mimic drug-induced injury [31, 32].

Here we present an overview of some cardiovascular lesions that spontaneously occur in the species most commonly utilized in animal research and toxicity testing. Special emphasis has been given to the spectrum of spontaneous background myocardial pathology that might be encountered during preclinical studies initiated to identify potential cardiotoxic actions of anticancer agents.

### Mice

#### Rodent progressive cardiomyopathy

Evidence of spontaneous cardiomyopathy has been reported in at least two common strains of mice (BALBc/Cr and B6C3f1) [32–36]. Early morphologic changes consisted of myocyte degeneration and necrosis without concomitant inflammatory infiltrates and fibrosis. In some instances, the only discernible alteration was a few small, irregular foci of fibrosis [32–35], most often in the wall of the left ventricle and interventricular septum. The lesions were generally not severe but could have become so with age.

#### Myocardial mineralization

Myocardial mineralization has been reported in various inbred mouse strains (DBA/2, C, C3H, BALB/c, A, CBA, and CHI) [37]. Mineralization was detectable as early as 1 month after birth. The primary location varied by strain: the epicardium in BALB/c, the myocardium in C3H and C3Hf, and the epicardium and myocardium of the right ventricle in DBA/2 [37]. These mineralization’s have been called several names, with dystrophic cardiac calcinosis or mineralization being the most common. The frequency and severity of mineralization in these animals can be influenced by age, sex, diet, and number of pregnancies [37–40]. The cause has been ascribed to focal myocyte necrosis [37], spontaneous myocarditis [41], and innate immunologic activity [39]. Macroscopically, some instances of epicardial and myocardial mineralization appear as multiple small, white-to-yellow specks [37]. Microscopically, areas of mineralization can contain prominent eosinophilic infiltrates [41]. Calcified lesions in BALB/cByJ mouse hearts also included immune cells, collagen fibers, and degenerating myocytes [39]. Myocardial mineralization can possibly occur as a result of alterations in other organs such as the kidney [30].

#### Atrial thrombosis

Atrial thrombosis occasionally occurs spontaneously in certain strains of mice (BALB/c, TS, RF, C, DBA) [37, 42]. The atria appear grossly swollen, firm, and mottled. A gray-to-tan thrombus in these atria can vary from recently formed layers of thrombin to older, organized thrombi with fibrous connective tissue [37]. Some thrombi may extend into the orifice of the mitral valve. Thrombi appear to develop more often in the left atria but can occur in both atria [37]. In mice, atrial thrombi can be influenced by heredity, sex, age, diet, and number of pregnancies [37]. Atrial thrombosis has also been observed in mice treated with the antineoplastic agent, doxorubicin [13, 43].

#### Vascular injury

Spontaneous systemic vascular injury (synonyms for vascular injury include vasculitis, arteritis, polyarteritis, periarteritis, and necrotizing arteritis) occurs in both inbred and outbred strains of mice. Vascular injury is a conspicuous morphologic finding in most murine models susceptible to autoimmune disorders (MRL/lpr, NZB, NZB/W, BXSB, and SNF1) [44]. In MRL/lpr mice, inflammatory vascular lesions spontaneously develop in lymphoid and other tissues [44]. These lesions are located mainly in small and medium-sized muscular arteries and are described as a necrotizing arteritis or necrotizing polyarteritis with a component of fibrinoid necrosis [45]. Initially, neutrophilic granulocytes accumulate near necrotic vessel walls. Subsequently, histocytes, lymphocytes, and fibroblasts appear in the medial and adventitial portions of the affected vessels. The lesions can be present in many tissues but are most consistently found in the kidney and urinary bladder. The necrotizing vasculitis in MRL mice is morphologically similar to the polyarteritis nodosa in humans with systemic lupus erythematosus [45].

Spontaneous vascular injury has been observed in 10 to 55% of BALBc mice [46], where it is mainly confined to the base of the aorta. The prevalence can be affected by diet, sex, and age [30]. The incidence of arterial lesions (necrotizing polyarteritis) was associated with elevated blood pressure in eight inbred strains and one random-bred group of mice [47].

### Rats

#### Rodent progressive cardiomyopathy

Rats are susceptible to several naturally occurring myocardial degenerative alterations (myocyte degeneration or necrosis, inflammation, and fibrosis) that meet the definition of cardiomyopathy. These spontaneous alterations occur at varying degrees of incidence and severity in common strains of rats [31, 32, 35, 48, 49].

Spontaneous progressive cardiomyopathy was first thought to appear in rats that were at least one year of age [50]. However, spontaneous myocardial lesions have been detected in animals as young as 3 months old [48, 51]. These lesions tend to be more prevalent in hypertensive rats, in which they become more severe with age, and the incidence is higher in males than females [32, 51]. Morphologic changes typically follow a pattern that begins with focal to multifocal myocyte degeneration and necrosis, varying degrees of inflammation and interstitial cell infiltration, and ultimately, fibrosis. Inflammatory lesions are more common in younger rats, whereas fibrotic lesions are more common in older ones [32, 35, 48]. The most common sites of these spontaneous myocardial alterations reported by Chanut, et al. were the left ventricle, right ventricle and septum [48] and the papillary muscles, the subendocardial areas of the left ventricle, and the septum [32, 35, 51].

#### Left ventricular hypertrophy

Left ventricular wall thickness and left ventricular chamber size can vary between strains. For example, wall thickness was increased, and chamber size decreased in hearts from Sprague-Dawley compared to hearts from Lewis rats [49]. These differences appear related to myocyte dimensions as myocyte cell size in hearts from Sprague-Dawley rats are larger than those in Lewis rats [49]. Left ventricular hypertrophy was detected in 38% of the 104 Sprague-Dawley rats and 13% of the 64 Lewis rats studied. Spontaneous left ventricular hypertrophy occurred in animals that were less than 3 months old.

#### Endomyocardial fibrosis

Rats appear to be susceptible to a proliferative form of left ventricular subendocardial fibrosis [52]. Macroscopically, the affected subendocardial surface appears white and thickened. Microscopically, the lesion consists of a proliferation of fibroblast-like cells that are often confined to, or that are more severe in, the left ventricle [37, 52]. The incidence of endomyocardial lesions is 1 to 7% in several strains of rats and is higher in older animals [37].

#### Myocardial mineralization

Mineral deposition has been reported to occur in association with aging in many strains of mice and rats, most frequently following injury to the myocardium [30]. Myocardial mineralization has been observed to commence by 6 months of age in Fisher rats [53]. Myocardial mineralization can occur simultaneously with progressive cardiomyopathy or as a result of advanced renal disease [30]. The initiating factor might involve a systemic calcium-phosphorus imbalance [30].

#### Necrotizing Vasculitis

Spontaneous vascular alterations with varying incidence and anatomic distribution have been described in certain strains of rats, including spontaneous hypertensive rats [54]. This vascular lesion is more prevalent in males and appears as a degenerative necrotizing arteritis and polyarteritis nodosa that affects the small and medium-sized arteries in a variety of tissues, especially the mesentery, testes, and pancreas [30]. The pathogenesis of the necrotizing vasculitis has not been defined but might be initiated by immune complex deposition [54]. Characteristics include focal fibrinoid necrosis associated with inflammation, endothelial proliferation, and disruption or duplication of the elastic lamina [30]. The incidence of these vascular alterations can be influenced by diet, sex, age, and arterial pressure [30].

### Dogs

#### Chronic Valvular disease

Chronic valvular heart disease, also referred to as endocardiosis or myxomatous valve degeneration, is a common myocardial lesion in dogs [55, 56]. The atrioventricular valves, especially the mitral valve fibrosa, are most often affected [55–59]. Male and smaller breeds of dogs are more susceptible [57]. The cellular constituents and intracellular matrix of the valvular apparatus (valve leaflets and chordate tendineae) are altered. Initially, fibroelastic proliferation causes mild nodular thickening of the leaflets [58]. The severity of nodular thickening, which can occur in both the mitral and aortic valves, increases with age and is thought to be caused by the normal hemodynamic action of the heart. In most instances, these alterations are not clinically important [58]. A more ominous change is mucoid degeneration of the valvular leaflets. Water and stainable mucopolysaccharides accumulating on leaflet cells is an early indication of this lesion [56]. Over time, degenerative changes can result in ballooning of the cusp, mitral incompetence, and clinically important congestive heart failure [58]. The incidence of this disease is thought to increase with age. However, early valvular changes have been observed in 1-year-old dogs and in both young and adult beagle dogs [56, 60]. The causes and pathogenesis of spontaneous chronic valvular lesions in dogs is not fully understood.

#### Dilated cardiomyopathy

Spontaneous dilated (congestive) cardiomyopathy has been observed in dogs [61], more commonly in large-breed, middle-aged, male dogs [37]. The primary characteristics are ventricular dilatation and systolic pump failure [37]. The most common functional abnormalities are a rapid and irregular heart rate and congestive heart failure [61, 62]. Clinical signs include ascites, weight loss, weakness, and cough [62]. Macroscopically, the four chambers of the heart are dilated and enlarged. The ventricular walls are thin and contain small atrophic papillary muscles [62]. Microscopically, scattered foci of necrosis (especially near the left ventricular papillary muscles) and fibrosis can be observed [62]. In many cases, the factors provoking dilated cardiomyopathy are not obvious.

#### Cardiac hypertrophy

Cardiac hypertrophy occurs occasionally in various breeds of dogs and may be present without clinical signs [37]. This condition occurs most commonly in male dogs [36]. Necropsy findings in 10 dogs with naturally occurring cardiac disease closely resembled hypertrophic cardiomyopathy in humans [63]. Microscopic findings included ventricular hypertrophy, decreased left ventricular cavity size, and left atrial dilatation [63–65]. Symmetric septal hypertrophy was a common finding [64, 65].

#### Myocardial inflammation and necrosis

Spontaneously occurring necrotic lesions have been observed in the myocardia of beagle dogs. In 50 dogs ranging from less than 1 year to more than 5 years old, foci of degenerate or necrotic myocytes were found in 9 [66]. In 160 healthy control beagle dogs 9 to 20 months old, small areas of myocardial inflammation were found in 5% of males and in 2% of females [67]. Other alterations, which were not severe, included myxomatous (cartilaginous) changes in the cardiac skeleton (at the base of the heart) and variable degrees of Purkinje fiber vacuolation. Spontaneous myocardial lesions in control animals were not confined to any specific region of the heart [30, 67, 68].

#### Spontaneous vascular lesions

Background vascular lesions have been noted in healthy, non-treated beagle dogs [30]. The main morphological categories of spontaneous arterial lesions are degenerative, proliferative, or inflammatory [69]. In many instances, the frequency and severity of degenerative and proliferative arterial lesions increase with age or with diseases, such as those caused by infectious agents [69, 70]. However, acute and chronic systemic polyarteritis can occur spontaneously with no apparent cause. This type of vascular lesion has also been called idiopathic arteritis, polyarteritis, periarteritis, panarteritis, systemic vasculitis, perivascular vasculitis, necrotizing vasculitis, beagle pain syndrome, and idiopathic febrile necrotizing arteritis [70].

Beagle pain polyarteritis syndrome affects the small-to-medium muscular arteries in several organs, including the heart [70]. The most commonly affected cardiovascular site is the right coronary artery [70, 71]. Acute vascular changes range from histiocytic-lymphocytic periarterial infiltration to transmural neutrophilic inflammation with fibrinoid necrosis [71]. Subacute and chronic lesions show intimal hyperplasia with varying levels of ruptured internal elastic laminae and perivascular inflammatory cell infiltration [71]. Necrotic or inflammatory foci have also been noted in small, stenotic, intramyocardial arteries, as well as at sites where vessel blockage restricted blood flow into large branches of the coronary arteries [55].

The incidence of spontaneous arteritis in beagle dogs ranges from 3% to more than 30% [72, 73]. In another study, healthy control dogs had a very low incidence (1/103 and 1/98 in male and female dogs, respectively) of coronary artery alterations [67]. The incidence of arteritis appears to be slightly higher in males [73, 74].

### Miniature pigs

#### Cardiomyopathy

The incidence of spontaneous hypertrophic cardiomyopathy is 5 to 23% in several breeds of pigs [75]. The condition in pigs is in many ways morphologically and biochemically similar to that in humans [76]. These similarities include increased heart weight, thickening of the left and right ventricular free walls and septum (increased collagen matrix), disorientation of myocytes, myocardial fibrosis, and abnormalities in intramural coronary arteries [75, 77].

Congestive cardiomyopathy has also been observed in pigs [78]. Affected animals had concomitant conditions, such as aortic stenosis, pericarditis, and endocarditis, which may have contributed to a congestive cardiomyopathy-like syndrome. A similar type of spontaneous cardiomyopathy has not been detected in Göttingen miniature pigs [79].

#### Myocardial lesions

Spontaneous cardiac lesions in Göttingen mini pigs are rare. A comprehensive cardiac evaluation of 835 untreated control Göttingen mini pigs found one animal, or at most only a few, with myocarditis, inflammatory necrosis, pericarditis, epicardial or subepicardial edema, focal fibrosis, hemorrhage, arteritis or periarteritis, focal mineralization, or focal mononuclear cell infiltration [79].

#### Vascular lesions

Older pigs are prone to atherosclerosis (mainly in the elastic arteries and medium-to-large muscular arteries, such as the coronary arteries) [37]. Several inflammatory diseases affecting pigs are associated with an arteritis or vasculitis syndrome. This syndrome begins as acute necrosis of the tunica media (in the arteries and small arterioles) and progresses to thrombosis and infarcts in a variety of organs [80]. Background vascular alterations in healthy Göttingen mini pigs are generally focal and mild [79, 81]. Spontaneous arteritis in the small-to-medium arterioles and arteries has been detected in Göttingen mini pigs with idiopathic thrombocytopenia [80]. In some instances, these lesions had progressed to necrosis of the tunica media, thrombosis, and concentric laminar thickening of vascular walls that caused myocardial infarction [80].

### Monkeys

#### Myocardial alterations

Spontaneous myocardial alterations have been observed in the hearts from untreated control monkeys (cynomolgus, marmoset, and rhesus) [67, 82, 83]. The two most commonly reported myocardial alterations are focal inflammatory cell infiltrate (minimal to mild) and focal myocarditis (minimal to moderate). The inflammatory infiltrate consists of single or multiple aggregates of mononuclear cells (mainly lymphocytes) scattered throughout the myocardium (mainly the interstitium, perivascular spaces, or subepicardial or epicardial fat tissue) and that appeared to be unrelated to any specific myocyte injury [67, 82, 83]. Focal myocarditis, a second, less-common lesion, was characterized by mild-to-moderate aggregates of mixed inflammatory cells (granulocytes, macrophages, and lymphocytes). Focal myocarditis occurred at sites of myocyte injury (necrosis, karyomegaly, fibrin deposition). Both the inflammatory cell infiltration and the focal myocarditis were primarily in the subendocardial and subepicardial areas of the heart, including the base of the dorsal papillary muscle [82, 83].

Anisokaryosis and karyomegaly of cardiac myocyte nuclei have been found primarily in the left ventricle and intraventricular septum. Most of these nuclear changes were not prominent [67, 84].

Spontaneously occurring myocardial cell necrosis has been detected in experimental (20%) and breeding primates (30%) (*Macaca nemestrina* and *Macaca fascicularis*) [85]. Morphologic changes included multifocal areas of myocardial necrosis with concurrent lymphocytic infiltration. Both acute and chronic necrotic lesions were present, implying that the pathogenic process was either continuous or involved multiple components. This type of lesion might be induced by the stress-associated release of catecholamines that can occur with routine handling of the monkeys during experimental procedures [86]. The histologic characteristics of the lesion were similar to the necrotic alterations reported in monkeys after catecholamine administration [87].

The impact of the geographic source of cynomolgus macaque on differences in spontaneous cardiac pathology and response to xenobiotics revealed a novel spectrum of cardiac findings in Mauritian-source animals that had not been observed in Indonesian-source cynomolgus macaques [88]. When compared to predominantly Indonesian macaques, a higher incidence of myocardial degeneration was observed with additional novel findings including macroscopic and microscopic subendocardial hemorrhage with hemosiderin, myocardial fibrosis, and arterial medial degeneration/hemorrhage. Other findings including inflammatory cell infiltrates, anisokaryosis, and squamous plaques were observed with a comparable incidence as reported in Indonesian macaques [67]. Myocardial degeneration, subendocardial hemorrhage, and myocardial fibrosis can mimic test-article-related cardiac toxicity; therefore, a thorough understanding of the incidence and severity of spontaneously occurring cardiac lesions is necessary to prevent misidentifying test-article-related cardiac findings in different genetic sources of cynomolgus macaques in nonclinical safety testing.

Alterations such as myocardial mineralization, endocarditis, pericarditis, myocardial fibrosis, extramedullary hematopoiesis, squamous plaques, squamous epithelial plaques, and ectopic thyroid tissue have a low incidence in monkeys [67, 82, 83]. Spontaneous myocardial lesions have also been reported in baboon and chimpanzee hearts [89, 90].

#### Vascular lesions

Spontaneous vascular lesions have been reported in healthy monkeys. The accumulation of mucopolysaccharides (without lipid accumulation) in the intima of the aorta or coronary arteries (cynomolgus, marmoset, and rhesus) was a common incidental finding in untreated control cynomolgus monkeys [82]. The incidence of coronary arteritis was reported to be minimal in cynomolgus monkeys (0.5%) [82, 91].

## Conclusions

Differentiating drug-induced structural changes in cardiovascular tissues from naturally occurring cardiovascular lesions in laboratory animals is essential in preclinical safety testing of new drugs. We hope this review will raise awareness of spontaneously occurring cardiovascular lesions observed in commonly used laboratory animals and aid pathologists and toxicologists in their safety assessments during drug development.
